# IL-27 promotes NK cell effector functions via Maf-Nrf2 pathway during influenza infection

**DOI:** 10.1038/s41598-019-41478-6

**Published:** 2019-03-21

**Authors:** Pawan Kumar, Kamalakannan Rajasekaran, Arash Nanbakhsh, Jack Gorski, Monica S. Thakar, Subramaniam Malarkannan

**Affiliations:** 1 Laboratory of Molecular Immunology and Immunotherapy, Blood Research Institute, 8727 Watertown Plank Road, Milwaukee, WI 53226 United States; 2 Laboratory of Molecular Genetics, Blood Research Institute, 8727 Watertown Plank Road, Milwaukee, WI 53226 United States; 3 Departments of Pediatrics, Medical College of Wisconsin, Milwaukee, WI 53226 United States; 4 Departments of Microbiology & Immunology, Medical College of Wisconsin, Milwaukee, WI 53226 United States; 5 Departments of Medicine, Medical College of Wisconsin, Milwaukee, WI 53226 United States

## Abstract

Influenza virus targets epithelial cells in the upper respiratory tract. Natural Killer (NK) cell-mediated early innate defense responses to influenza infection include the killing of infected epithelial cells and generation of anti-viral cytokines including interferon gamma (IFN-γ). To date, it is unclear how the underlying cytokine milieu during infection regulates NK cell effector functions. Our data show during influenza infection myeloid cell-derived IL-27 regulates the early-phase effector functions of NK cells in the bronchioalveolar and lung tissue. Lack of IL-27R (*Il27ra*^−/−^) or IL-27 (*Ebi3*^−/−^) resulted in impaired NK cell effector functions including the generation of anti-viral IFN-γ responses. We identify CD27^+^CD11b^+^ NK cells as the primary subset that expresses IL-27R, which predominantly produces IFN-γ within the upper respiratory tract of the infected mice. IL-27 alone was incapable of altering the effector functions of NK cells. However, IL-27 sensitizes NK cells to augment both *in vitro* and *in vivo* responses mediated via the NKG2D receptor. This ‘priming’ function of IL-27 is mediated partly via transcriptional pathways regulated by Mafs and Nrf2 transcriptionally regulating TFAM and CPT1. Our data for the first time establishes a novel role for IL-27 in regulating early-phase effector functions of NK cells during influenza infection.

## Introduction

Each year thousands of people are hospitalized due to complication related to influenza virus infections. Innate and adaptive immune cells mediate the host immune responses to influenza virus infections. NK cells provide the first line of innate defense against influenza virus by killing infected epithelial cells and by producing anti-viral cytokine interferon (IFN)-γ^1,2^. NK cells express the multiple activating and inhibitory receptors to execute anti-viral or anti-tumor effector functions^3^. Virally-infected cells express H60, Rae, and Mult1 or Hemagglutinin (HA) ligands for NK cells activating receptor NKG2D and NCR1, respectively^4^. Recognition of ligands by NKG2D or NCR1 results in lysis of infected/tumor cells and the generation of IFN-γ from NK cells^5,6^. NK cells constitutively express or upregulate the expression of activating receptors to mount anti-viral responses; however, virally-infected/tumor cells evade NK cell-mediated recognition through various mechanisms. Virus down regulates ligands for NK cell-activating receptor or enhances engaging inhibitory receptors^4,7,8^.

Effect of cytokines in modulating NK cell responses has been an area of intense research. The common gamma receptor (γcR)-interacting cytokines IL-2, IL-7, IL-15, and IL-21 have been used to expand NK cells for adoptive transfer experiments in the clinical setting^9^. Unique α-chains define the receptors for these cytokines. IL-2 and IL-15 share a β-chain and the γcR along with cytokine-specific IL-2Rα and IL-15Rα, respectively^10,11^. Historically, IL-2 has been extensively used to expand murine and human NK cells^12,13^. IL-15 activates PI(3)K-mediated mTORC1 pathway^14,15^. IL-12 is a heterodimeric cytokine consists of p35 and p40 subunits, and it binds to the IL-12 receptor (IL-12Rβ1 and IL-12Rβ2)^16,17^. IL-18 belongs to an IL-1 family that interacts with a heterodimeric receptor composed of IL-18Rα and IL-18Rβ^18,19^. IL-12 and IL-18 enhance NK cell effector functions including IFN-γ production^20,21^. However, IL-12 or IL-18 responses are acute and independent of NK cell activating and inhibitory receptors^22^. IL-23 is another heterodimeric cytokine composed of p19 and p40 subunits, and its receptor is made up of IL-23Rα and IL-12Rβ1^23^. IL-23 activates NK cells to produce IL-22^24,25^. IL-35 contains p35 and EBI3 subunits, and its recently defined receptor consists of IL-12Rβ2 and gp130^26–28^. gp130 is the shared receptor subunit of an IL-6 family of cytokine receptors^29^. IL-27 is another heterodimeric cytokine that belongs to the IL-12 family and consists of p28 and Epstein–Barr virus-induced gene 3 (EBI3)^30^. Receptor for IL-27 is composed of gp130 and WSX1^31^.

IL-27 and has been shown to modulate NK cells anti-tumor cytotoxicity responses^32–35^. These studies demonstrate that IL-27 augments NK cells cytotoxic responses to a variety of tumor cell lines in perforin, granzyme, TRAIL, and Fc-γR-III-dependent mechanisms^32,33,36–39^. The role of IL-27 in NK cell-mediated anti-tumor immunity has been defined^39^. However, the underlying molecular mechanism is not well-defined. Notably, the mechanism by which IL-27 regulate NK cells effector functions during viral infections is yet to be fully understood.

In this study, we determined the role of IL-27 signaling in regulating NK cells effector responses during influenza infection as well as dissecting molecular mechanism of its action. Our data show that NK cells upregulate IL-27R following influenza infection. IL-27 but not IL-12 or IL-35 is obligatory for promoting the early NK cell-mediated responses. *Ebi3*^−/−^ and *Il27ra*^−/−^ mice exhibited significantly reduced NK cell effector functions (IFN-γ and cytotoxicity) during influenza infection. Our *in vivo* and *in vitro* findings strongly suggest that defect in effector responses were NK cells intrinsic and involve CD27^+^CD11b^+^ subset. Mechanistically, IL-27 regulates NK cells effector functions via small Maf-F and Nrf2. Expressions of γ-glutamylcysteine ligase catalytic (GCLC), mitochondrial transcription factor A (TFAM), and carnitine palmitoyltransferase 1 (CPT1) were significantly reduced in NK cells derived from *Ebi3*^−/−^ mice, demonstrating a unique and exclusive functional role for IL-27. These findings provide a novel insight into how IL-27 plays a central role in containing viral infections by sensitizing NK cells to recognize infected cells and provide protective immunity to the host.

## Results

### Influenza infection leads to IL-27 generation and induction of IL-27R

**Figure 1:**
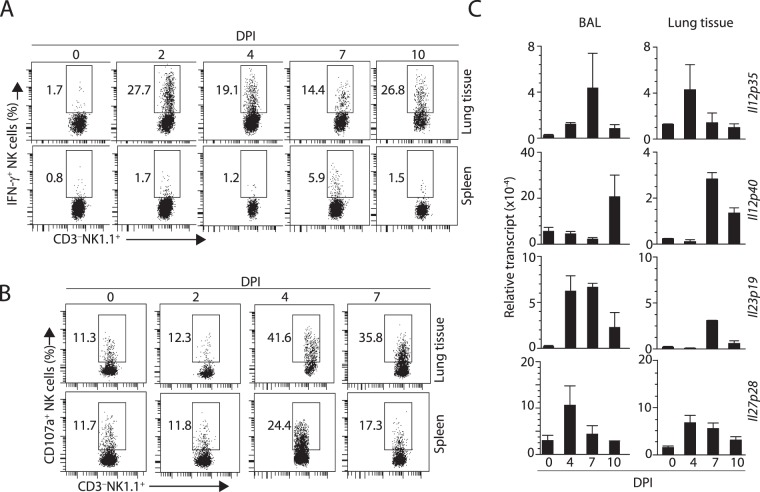
NK cell infiltration and function coincides with IL-27p28 expression in the BAL and lung tissue of mice during the early phase influenza infection. (**A**) Production of IFN-γ peaks on DPI 4. Intracellular IFN-γ was analyzed in gated the lung tissue or spleens from the infected mice on indicated DPIs. (**B**) Expression of CD107a, a surrogate marker for the release of cytolytic granules including granzyme B peaks on DPI 4 in both lung tissue and spleens of the infected mice. (**C**) Expression of *Il12p35*, *Il12p40*, *Il23p19*, and *Il27p28* transcripts in the BAL cells and lung tissue on different DPIs. Data shown are from two or three independent experiments with 4–7 mice (**A**), 3–4 mice (**B**) or 4 mice (**C** except for DPI7). DPI = Days post infection

Activation of innate and adaptive immune responses is natural host defense against influenza infection. The innate immune response includes infiltration of myeloid cells and NK cells in the upper respiratory tract. To determine the functional relevance of NK cells during influenza infection, we intranasally infected C57BL/6 (wild-type, WT) mice with 500 PFU of mouse-adapted human A/PR/8/34 H1N1 (PR8) influenza virus. The lumen side of the trachea consists of a thick layer of columnar epithelial cells that are exposed to and are the targets of influenza virus. Our earlier study has shown that effector lymphocytes including NK cells infiltrate into the epithelial layer via the basement membrane^40^. We found only fewer NK cells were present on days post-infection (DPI) 0 and their number steadily increased on DPI 4 and 7. Our earlier work has shown that NK cells are abundantly present within the alveolar space and conducting airways of the lungs during influenza infection^5,40–42^. To define the functional relevance, we analyzed the production of IFN-γ by NK cells on DPI 0, 2, 4, 7, and 10. Percentages of IFN-γ^+^ NK cells considerably increased on DPI 2 and DPI 4 (Fig. 1A. Expression of CD107a (LAMP1), which is a surrogate marker for cytotoxicity peaked on DPI 4 (Fig. 1B).

Inflammatory cytokine milieu within the infected trachea and alveolar space regulate the production of IFN-γ from NK cells during influenza infection. To identify the relative contribution of IL-12, IL-27, and IL-23, we infected WT mice with influenza virus. Single cell suspensions from bronchoalveolar lavage (BAL) and the lung tissues were used to quantify the transcript levels of *Il12p35*, *Il12p40*, *Il23p19*, *and Il27p28*. Cells from both BAL and lung tissues contained transcripts encoding these cytokines. We found *Il27p28* transcripts consistently appeared earlier (DPI 4) than *Il12p35* and *Il12p40* or *Il23p19* (DPI 7) in both BAL and lung tissues (Fig. 1C).

**Figure 2:**
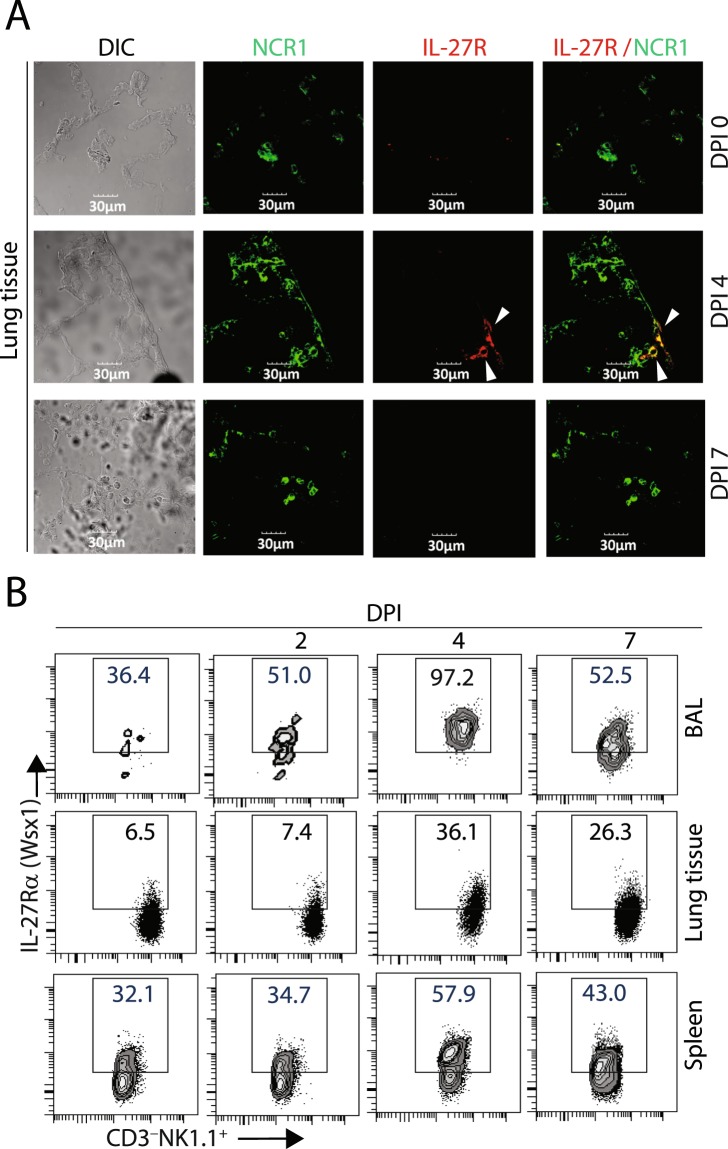
Expression of IL-27R (Wsx-1) on NK cells during influenza infection. (**A**) Confocal analyses of IL-27R expression in NCR1^+^ NK cells in the lung tissue on indicated DPIs. Lung sections from infected mice were stained with anti-NCR1 and anti-Wsx-1 mAbs. (**B**) Flow cytometric analyses of IL-27R expression among CD3^−^NK1.1^+^ NK cells obtained from BAL, lung tissue, or spleens of influenza-infected mice on different days of post-infection. Percent IL-27R (Wsx-1) positive cells are shown. Three mice each for A and B are analyzed, and representative images or data are presented.

We next analyzed the expression of the IL-27 receptor (IL-27R) temporally during influenza infection. The IL-27R is composed of IL-27Rα (Wsx1) and the shared IL-6 family receptor subunit, gp130. To determine its expression, we used an antibody specific for IL-27Rα (Wsx1). We performed confocal analyses of NK cells from lung tissues (Fig. 2A) and flow cytometry analyses (Fig. 2B) of NK cells from BAL, lung tissues, and spleen of infected mice from different DPIs. Our data reveal expression of IL-27Rα in NK cells peaked on DPI 4 coinciding with the production of IL-27. Next, we characterized the cell type that produces the IL-27. Cells from both BAL and lung tissues were stained define Ly6G^+^ cells myeloid population as the predominant producer of IL-27 during this early period of influenza infection (Supplementary Fig. 1A and B). Consistent with published data, production of IL-27 by Ly6G-positive cells temporally aligned with the expression of IL-27R on NK cells during influenza infection^43^.

### IL-27 augments NKG2D but not IL-12-mediated IFN-γ production

**Figure 3:**
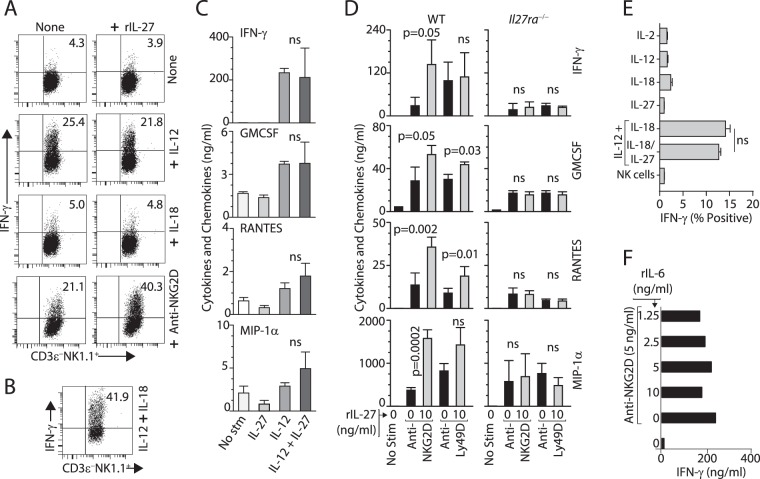
IL-27 regulates NKG2D and Ly49D-mediated effector functions of NK cells. (**A**) IL-27 augments NKG2D- but not IL-12-mediated IFN-γ production. NK cells were cultured with IL-12 (10 ng/ml), IL-18 (10 ng/ml), or plate-bound anti-NKG2D mAb (5 ng/ml) for 12 hours and the production of IFN-γ was examined by intracellular staining. (**B**) IL-12 (1 ng/ml) in combination with IL-18 (10 ng/ml) stimulate NK cells to produce a significant amount of intracellular IFN-γ. (**C**) IL-27 regulates the production of multiple cytokines and chemokines. NK cells from WT mice were stimulated with IL-27 (10 ng/ml), IL-12 (10 ng/ml), or IL-27+ IL-12 (10 ng/ml each) for 18 hours and the supernatants were tested for the presence of indicated cytokines and chemokines. (**D**) Activation receptor-mediated inflammatory cytokine and chemokine production are regulated by IL-27. NK cells from WT or *Il27ra*^−/−^ were stimulated with anti-NKG2D (5 ng/ml) or anti-Ly49D mAb (5 ng/ml) in the presence or absence of rIL-27 (10 ng/ml). (**E**) IL-27 does not mediate additive effect on IL-12 and IL-18-mediated IFN-γ production in NK cells. Purified NK cells were cultured with indicated individual or combinations of cytokines (10 ng/ml each) for 12 hours and the IFN-γ was examined by intracellular staining among CD3ε^−^NK1.1^+^ NK cells. (**F**) rIL-6 do not play a similar role as that of IL-27. Titrated concentrations rIL-6 (1.25 to 10 ng/ml) were used in the presence of mitogenic anti-NKG2D mAb (5 ng/ml). IL-2-cultured NK cells were used (**A**–**E**). Data presented are a representative of 3–4 independent experiments generated from 3–4 mice per group per experiment.

Proinflammatory cytokines IL-12 and IL-18 can stimulate the production of IFN-γ. IL-23 is primarily responsible for the production of IL-22 from effector lymphocytes including NK cells^40,44,45^. Generation of IL-12 during the early phase of influenza infection indicates that these cytokines may play a role in regulating the IFN-γ gene transcription in NK cells during the early phase of infection. Influenza infection also leads to the expression of inducible stress proteins such as H60, Rae-1, and Mult1 in infected cells that are the cognate ligands for the activation receptor NKG2D^46^. Infected epithelial cells also express viral haemagglutinin, which is one of the defined ligands of the activating NK cell receptor, NCR1 (Nkp46). To distinguish the role of IL-27 on activation receptor (such as NKG2D or Ly49D) and cytokine receptors (such as IL-12 or IL-18), we stimulated IL-2-cultured NK cells with combinations of stimuli (Fig. 3). Recombinant IL-27 (rIL-27) augmented only NKG2D- but not IL-12 or IL-18-mediated activation (Fig. 3A). Although IL-12 is a potent stimulator of IFN-γ, the presence of exogenous IL-27 did not augment IFN-γ production. In contrast, the presence of IL-18 along with IL-12 resulted in the maximal production of intracellular IFN-γ (Fig. 3B). These observations were further validated for other cytokines and chemokines including GM-CSF, RANTES, and MIP1-α by testing the culture supernatants via multiplex assays (Fig. 3C).

To further corroborate these findings, we stimulated IL-2-cultured splenic NK cells from WT and *Il27ra*^−/−^ mice with anti-NKG2D or anti-Ly49D mAbs in the presence or absence of recombinant IL-27. IL-27 alone was not able to induce the generation of IFN-γ (Fig. 3D). However, IL-27 significantly augmented anti-NKG2D mAb-mediated production of cytokines and chemokines in NK cells from WT mice. Role of exogenous IL-27 on anti-Ly49D mAb-mediated generation of GM-CSF and RANTES was significant; however, its effect on IFN-γ and MIP-1α was only moderate in NK cells from the WT. Importantly, the addition of rIL-27 did not have any effect on NK cells from *Il27ra*^−/−^ mice confirming the specific role of IL-27 on activation receptor-mediated cytokine and chemokine production (Fig. 3D). IL-12 and IL-18 together promote optimal production of IFN-γ. rIL-27 along with IL-12 did not significantly augment the production of IFN-γ. Therefore, we next tested whether the combination of IL-12, IL-18, and IL-27 has an additive effect on NK cells. There were no significant differences between IL-12 and IL-18 or the combination of all three cytokines in the production of IFN-γ production (Fig. 3E). These observations confirm that IL-27 plays a central role in the co-stimulation activation receptor (such as NKG2D)-mediated NK cell effector functions.

IL27R and IL-35R belong to the IL-6R family, and they utilize a common receptor chain, gp130 (Ref). Also, to distinguish the unique role of IL-27 and its receptor IL-27R (IL-27Rα/gp130) from IL-6R in NK cells, we stimulated IL-2-cultured NK cells from WT mice with anti-NKG2D mAb in the presence of increasing concentrations of recombinant IL-6 protein (rIL-6). We found that in contrast to rIL-27, rIL-6 was unable to augment anti-NKG2D mAb-mediated activation and production of IFN-γ even at a higher concentration of 10 ng/ml (Fig. 3F).

### IL-27 but not IL-35 regulates NK cells effector function *in vivo*

**Figure 4:**
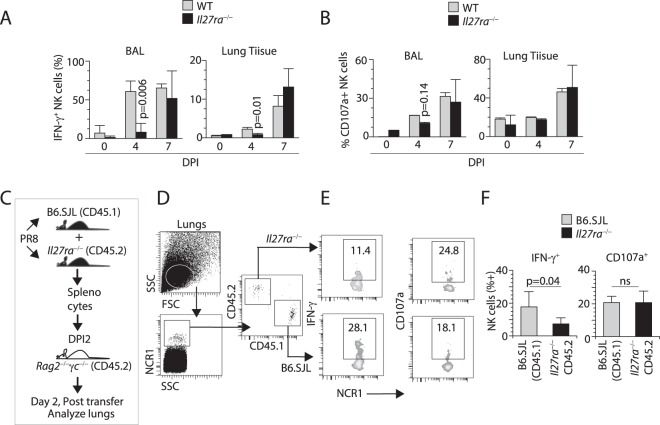
IL-27 regulates NK cells effector function during influenza infection. (**A**) Lack of IL-27Rα significantly reduces the percentages of intracellular IFN-γ^+^ NK cells. Bar diagram of IFN-γ^+^ NK cells from BAL and lung tissues of WT and *Il27ra*^−/−^ mice different DPI are shown. (**B**) Lack of IL-27Rα does not affect the cytotoxic potentials of NK cells as measured by its surrogate marker CD107a (Lamp1). NK cells from BAL and lung tissues of WT and *Il27ra*^−/−^ mice different DPI are shown. (**C**) The schematic for the adoptive transfer of splenocytes from WT (CD45.1) and *Il27ra*^−/−^ (CD45.2) mice into *Rag2*^−/−^*γc*^−/−^ mice. Host *Rag2*^−/−^*γc*^−/−^ mice were infected with influenza virus, and their splenocytes were analyzed on DPI 2. (**D**) Lungs from *Rag2*^−/−^*γc*^−/−^ mice isolated and the CD3^−^NCR1^+^ NK cells were analyzed. (**E**) Percent IFN-γ or CD107a (LAMP1)-positive NK cells from one representative mouse each are shown. (**F**) Average percent positive cells from four mice per genotype are shown. Data in A, B, D-F are averages with standard deviation and are obtained from a minimum of four individual mice and are representatives of at least two independent experiments.

IL-35 is a heterodimeric cytokine that shares the EBI3 subunit with IL-27 and p35 (IL-12a) with IL-12. IL-35 signals through a receptor composed of IL-12Rβ2 and gp130^28^. To differentiate the functional role of IL-27 from IL-12 and IL-35, we infected *Il27ra*^−/−^ (Wsx1^−/−^) or *Il12a*^−/−^ (*Il12p35*^−/−^) mice with influenza virus. Lack of IL-27Rα receptor subunit significantly reduced the levels of IFN-γ production in NK cells on DPI 4 (Fig. 4A) and a moderate reduction in LAMP1 expression (Fig. 4B). Alterations in the percent IFN-γ-positive NK cells were further validated by analyzing the absolute number of NK cells in both BAL and lung tissues (Supplementary Fig. 2A and B). NK cells from both *Il27ra*^−/−^ and WT generated comparable levels of IFN-γ on DPI 7 (Fig. 4A), indicating the possible role of other cytokines such as IL-12. In contrast to *Il27ra*^−/−^ mice, NK cells from the BAL or lung tissue of *Il12a*^−/−^ mice generated comparable levels of IFN-γ on DPI 4, to that of WT (Supplementary Fig. 3A–C). This suggests a unique role of IL-27 in augmenting NK cells responses during the early phase (DPI 0–4) of influenza infection. Importantly, NK cells from DPI 2 that express the IL-27R (Wsx-1) are the ones that also produced IFN-γ and granzyme B demonstrating a causal relationship between the expression of IL-27R and NK cell effector functions during influenza infection (data not shown).

To eliminate the possibility of an NK cell extrinsic defect in the observed effector functions in mice lacking IL-27R (*Il27ra*^−/−^) mice, we performed adoptive transfer experiments. Splenocytes from WT (B6.SJL, CD45.1^+^) and *Il27ra*^−/−^ (CD45.2^+^) mice were isolated, and a mixture (1:1) of these cells were adoptively transferred into lymphocytes-deficient *Rag2*^−/−^γ*c*^−/−^ mice on (Fig. 4C). Host mice were infected with influenza. On DPI 2, we were able to detect NCR1^+^ WT and *Il27ra*^−/−^ NK cells in the lung tissue of *Rag2*^−/−^γ*c*^−/−^ mice (Fig. 4D). The percentages of NK cells transferred from *Il27ra*^−/−^ mice that produced IFN-γ were significantly lower compared to that of B6.SJL (Fig. 4E,F). However, the overall percentages of LAMP1^+^ NK cells did not vary between the B6.SJL (CD45.1) and *Il27ra*^−/−^ CD45.2) mice. Collectively these data suggest IL-27 plays a primary role in the production of IFN-γ from NK cells during the early phase of influenza infection *in vivo*.

### IL-27 regulates CD27^+^CD11b^+^ effector NK cell population *in vivo*

**Figure 5:**
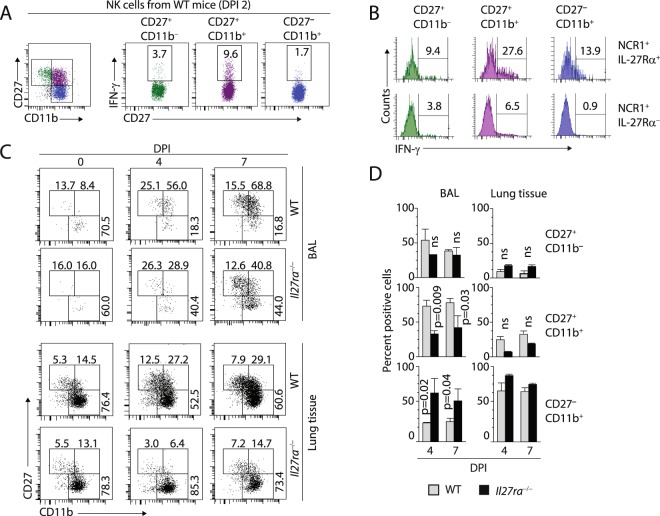
Lack of IL-27Rα results in reduced number of CD27^+^CD11b^+^ NK cells subset during influenza infection. (**A**) CD27^+^CD11b^+^ NK cell subset predominantly produces IFN-γ during influenza infection. CD3^−^NCR1^+^ NK cells from the lungs of influenza-infected WT mice on DPI 2 are sub-divided based on CD27 and CD11b (left), and the levels of IFN-γ^+^ was analyzed in each subset (right). (**B**) Among CD27^+^CD11b^+^ subset, IL-27Ra^+^ NK cells are the predominant producers of IFN-γ. NK cells from the lungs of WT mice on DPI 2 were analyzed based on CD27, CD11b, and IL-27Rα staining. (**C**) The number of CD27^+^CD11b^+^ NK cells are significantly reduced in the lungs of *Il27ra*^−/−^ mice during influenza infection. CD3^−^NCR1^+^ NK cells from BAL and lungs of WT and *Il27ra*^−/−^ mice on indicated DPI were stained for the expression of CD27 and CD11b. (**D**) Bar diagram shows the percentage of different effector NK subsets in the BAL and lungs of WT and *Il27ra*^−/−^ mice on DPI 4 and DPI 7. Data presented in A and B was a representative one mouse, which is a representative of 4–6 mice from two independent experiments. Data presented in D were an average of three mice per genotype.

Expression of CD27 on T cells along with CD44 marks the onset of early effector memory phenotype^47^. The absence of CD27 on CD44^+^CD62L^−^ T cells define the late effector memory and effector T cell subsets. In the case of NK cells, expression of CD27 and CD11b define functional subsets of NK cells^48,49^. CD27^+^CD11b^+^ and CD27^+^CD11b^−^ NK cells are specialized to secrete cytokines, whereas CD27^−^CD11b^+^ NK cells are more cytotoxic^48,49^. To identify the primary subset of NK cells that produces IFN-γ during the early phase of influenza infection, we analyzed the lungs of the WT mice on DPI 2. Lung-derived NK cells were gated based on their CD27/CD11b positivity (Fig. 5A). We found that the majority of NK cells that produced IFN-γ were the CD27^+^CD11b^+^ compared to the other two subsets. Next, we gated the NK cells from the lung of the infected mice (DPI 2) based on the expression of IL-27R into NCR1^+^IL-27R^+^ or NCR1^+^IL-27R^−^ cells. These two subsets were further divided into CD27^−^CD11b^+^, CD27^+^CD11b^+^, and CD27^+^CD11b^−^ NK cells and the production of IFN-γ examined. Among these, IL-27R^+^ CD27^+^CD11b^+^ NK cells were proportionately most positive for intracellular IFN-γ compared any other subsets (Fig. 5B). Also, among the IL-27R^−^ NK cells, CD27^+^CD11b^+^ NK cells were predominant in producing IFN-γ (Fig. 5B). Our data suggest that the CD27^+^CD11b^+^ NK cell subset may have an inherent maturation or recruitment defect in *Il27ra*^−/−^ mice.

Distinct stages of development and acquisition of unique functional receptors characterize NK cell maturation in the BM. CD27^+^CD11b^−^ subset represents the earliest stage of functional maturation of NK cell development in the BM^50,51^. These single positive subset transition into the CD27^+^CD11b^+^ intermediate stage and eventually into CD27^+^CD11b^−^ NK cells. CD27^−^CD11b^+^ NK cells were predominant in circulation as well as in the lung tissue^49^. Furthermore, CD27^+^CD11b^+^, as well as CD27^+^CD11b^−^ NK cells, have the more proliferative capacity^49,52^. Irrespective of this knowledge, it is not clear if NK cells subset specification changes under inflammatory condition. To define this, we analyzed the BAL and the lung tissues of influenza-infected WT and *Il27ra*^−/−^ mice on DPI 0, 4, and 7 (Fig. 5C,D). We found a significant reduction in the percentages of CD27^+^CD11b^+^ NK cell effector population in the alveolar space of *Il27ra*^−/−^ but not WT in influenza-infected mice. Also, there was a proportionate and a concomitant increase in the percentages of CD27^−^CD11b^+^ NK cell subset at an earlier stage of influenza infection. The changes in the percentages of these subsets were not due to a change in the absolute number of NK cells in the BAL of the infected mice. Absolute number of NK cells per million lymphocytes in the BAL ranged between 17,400 to 35,550 in the WT on DPI4. In mice that lacked IL-27Rα, it ranged between 11,500 to 20,000 NK cells. On DPI7, it was between 62,400 to 123,250 in the WT mice while in *Il27ra*^−/−^ mice between 128,250 to 179,550. CD27+ CD11b−, CD27+ CD11b+, CD27− CD11b+ NK cells were distributed as shown with the percentages. Thus, in fact the absolute number of NK cells in the BAL during influenza infection between the WT and the *Il27ra*^−/−^ mice were either comparable or moderately increased in the *Il27ra*^−/−^ mice. We next examined the bone marrow (BM) of naïve mice for these different NK cell subsets. Interestingly, *Il27ra*^−/−^ mice have significantly less CD27^+^CD11b^+^ NK cells in the BM, suggesting IL-27 may have a role in NK cells development or maturation or proliferation (Supplementary Fig. 4A and B). Our data further show a similar level of various inhibitory and activating NK cell receptors in naïve BM-derived WT and *Il27ra*^−/−^ mice, suggesting a more specific role of IL-27 in regulating CD27^+^ NK cell subsets (Supplementary Fig. 4C–E).

### Lack of EBI3 reduces the ability of NK cells to produce IFN-γ during influenza infection

**Figure 6:**
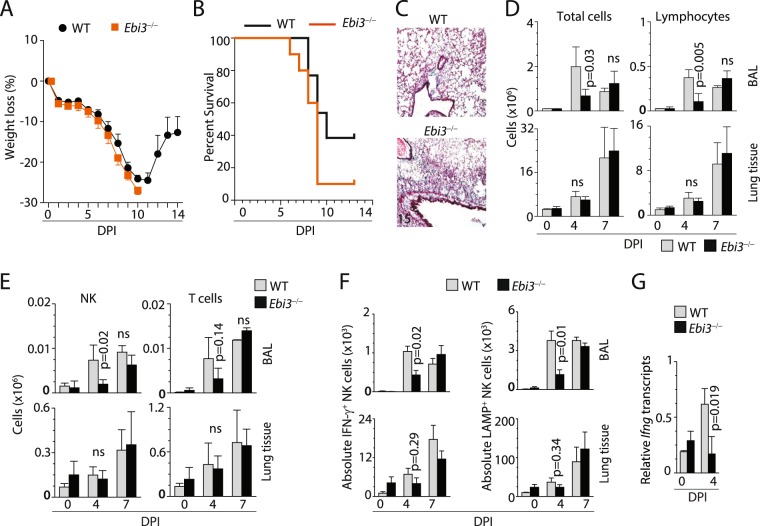
Mice lacking EBI3 exhibit significantly impaired NK cell-mediated effector functions. (**A**) Mice that lack EBI3 do not recover their weight loss and succumb to influenza infection. WT and *Ebi3*^−/−^ mice were infected with influenza virus, and their weight loss or (**B**) survival was monitored over the indicated DPIs. (**C**) Lack of EBI3 leads to increased inflammation as characterized by collagen deposition in the lung tissue (Mason’s trichrome staining, blue). (**D**) Lack of EBI3 decreases the total number of lymphocytes recruited to the lung. (**E**) Total number of NK cells recruited to the lungs were decreased in mice lacking EBI3. (**F**) An absolute number of NK cells producing IFN-γ or expressing CD107a (LAMP1) were significantly decreased in the absence of EBI3. (**G**) The reduction in the production of IFN-γ is due to an impaired transcription of *Ifng* gene. NK cells were isolated on indicated DPIs from the lungs of WT and Ebi3^−/−^ mice, total RNA was isolated, and used to quantify the abundance of the transcripts. Data were generated from two independent experiments with 8–10 (**A**), 3–6 (**B**), 3–5 (**C**,**D**) or 3–4 (**E**, except DPI 0) mice per group.

IL-27 cytokine consists of two subunits, EBI3, and IL-27p28. To further confirm the role of IL-27 in regulating NK cells effector functions *in vivo*, we infected WT and *Ebi3*^−/−^ (the critical subunit of both IL-27 and IL-35 cytokines) mice with influenza virus. NK cells upregulate IL-27R in WT mice in response to influenza infection (Fig. 2A,B). Our data suggests *Ebi3*^−/−^ mice are more susceptible to influenza infection as revealed by weight loss (Fig. 6A), mortality curve (Fig. 6B) and increased collagen deposition in the lung tissue as detected by Masson’s trichrome staining (Fig. 6C). Interestingly, lymphocytes (Fig. 6D) and specifically NK and T cells (Fig. 6E) recruitment to the bronchoalveolar space (BAL), but not lung tissue/parenchyma were significantly reduced in *Ebi3*^−/−^ mice early point of infection. In line with these observations, NK cells in the bronchoalveolar space were not capable of mounting early-phase anti-viral effector functions as revealed by reduced intracellular IFN-γ in *Ebi3*^−/−^ mice at the early time point of infection (Fig. 6F). *Ebi3*^−/−^ mice displayed reduced IFN-γ production at the transcriptional level (Fig. 6G), suggesting IL-27 regulates NK cells responses at an early phase of influenza infection.

### IL-27 regulates NKG2D-dependent cytotoxicity

Our data suggest IL-27 enhance NKG2D-mediated cytokines generation. Earlier work suggested that IL-27-augmented cytotoxicity of NK cells is dependent on the upregulation of NKG2D ligands on the epithelial tumor and via ADCC^53^. We next examined the requirement of IL-27 in regulating NKG2D-mediated NK cells cytotoxicity. We used two stable EL4 cell lines (*induced-self*), RMA-S (*missing-self*) and YAC1 (*non-self*) in cytotoxicity assays. IL-2-cultured NK cells from *Ebi3*^−/−^ or *Il27ra*^−/−^ mice have shown similar cytotoxicity towards EL4-H60^low^, EL4-H60^high^, RMA-S, and YAC1 cell lines as compared to WT mice, suggesting there is no inherent defect in the cytotoxic potentials of NK cells (Supplementary Fig. 5A). We found that recombinant IL-27 enhances the cytotoxic capacity of NKG2D-mediated WT and *Ebi3*^−/−^ but not *Il27ra*^−/−^ mice IL-2-cultured NK cells (Supplementary Fig. 5B).

### IL-27-dependent activation of NF-κB, T-bet, MafF, and Nrf2 regulates NK cells effector function

**Figure 7:**
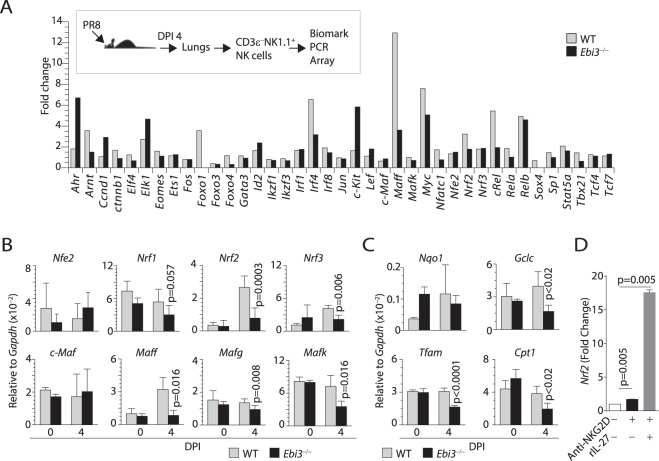
IL-27 regulates MAFF and Nrf2 expression during influenza infection. (**A**) NK cells from the influenza-infected WT and *Ebi3*^−/−^ mice on DPI 4 were analyzed for the expression levels of a panel of transcription factors using Fluidigm Transcription Factor arrays. (**B**) RT-qPCR data shows expression of *Nfe2*, *Nrf1*, *Nrf2*, *Nrf3*, *cMaf*, *Maff*, *Mafg*, and *Mafk* on sorted NK cells from WT and *Ebi3*^−/−^ mice on day four post influenza infection. (**C**) RT-qPCR data showing reduced expression of *Nqo1*, *Ho*, *Tfam1*, and *Cpt1* on sorted NK cells from WT and *Ebi3*^−/−^ mice on day four post influenza infection. (**D**) NK cells were stimulated with plate-bound anti-NKG2D mAb in the presence or absence of rIL-27 and the mRNA were analyzed for the expression levels of *Nrf2*. Gene array data were generated using Fluidigm 50-selected gene array from sorted NK cells of WT (n = 2) and *Ebi3*^−/−^ (n = 2) day four influenza infected or control mock-infected mice. Data in (**B**) and (**C**) generated from 4 mice per genotype (except mock-infected mice).

Our data strongly suggest a critical role of IL-27 in regulating NK cells effector functions *in vivo* and *in vitro*. We next investigated the molecular mechanisms by which IL-27 regulate the effector functions of NK cells. Towards this, we purified NK cells from WT, and *Ebi3*^−/−^ mice on DPI 4 following influenza infection, purified mRNA, and performed gene array analyses for a panel of 48 transcription factors using Biomark chips. We found transcript levels of *Ahr* (Aryl hydrocarbon receptor), *Ccnd1* (Cyclin D1), *Elk1* (ETS domain-containing protein), and *cKit* (CD117) were considerably increased (Fig. 7A). In contrast, *Foxo1* (Forkhead box 1), *Foxo4* (Forkhead box 4), *Irf4* (Interferon regulatory factor 4), *Myc* (bHLH transcription factor), *Nfatc1* (NF-AT), *cRel* (NF-κB subunit), *Rela* (NF-κB subunit), and *Tbx21* (T-bet) were substantially reduced (Fig. 7A). Among these, transcription factors such as c-Rel, Rel-a, T-bet are known to play a direct role in the transcription of IFN-γ and thus providing a mechanistic explanation of how IL-27 regulates cytokine production in NK cells. Also, our data showed a reduction in the expression of v-Maf musculoaponeurotic fibrosarcoma oncogene (Maf) homolog F (MafF) but not c-Maf in NK cells from *Ebi3*^−/−^ mice (Fig. 7A).

Nuclear factor E2–related factor 2 (Nrf2) binds to the ARE sequence as a heterodimer with one of the small bZIP proteins, Mafs, and activates specific gene transcriptions. We confirmed gene array data using RT-qPCR analyses of c-Maf, MafF, and MafK expression in sorted NK cells from influenza-infected WT and *Ebi3*^−/−^ mice (Fig. 7B). The gene array data also show that the expressions Nrf1 and Nrf2 were significantly reduced in *Ebi3*^−/−^ mice sorted NK cells (Fig. 7B), suggesting IL-27 stimulate NK cells through MafF/MafG-Nrf2 pathways. Nrf2 plays an indispensable role in augmenting the expression of Phase-II detoxifying and the anti-oxidant enzymes. Therefore, we investigated the expressions of quinone oxidoreductase (NQO1) and γ-glutamylcysteine ligase catalytic (GCLC). Although the transcript levels of *Nqo1* did not change, levels of *Gclc* was significantly reduced in NK cells from *Ebi3*^−/−^ mice (Fig. 7C). In addition, we found that transcripts encoding both mitochondrial transcription factor A (TFAM) and carnitine palmitoyltransferase 1 (CPT1) were also significantly reduced in NK cells from *Ebi3*^−/−^ mice compared to that of WT (Fig. 7C). Our study demonstrates that IL-27 has an augmenting effect on NKG2D-mediated IFN-γ production from NK cells (Fig. 3). To define whether Nrf2 gene was a direct target of this combined activation, we stimulated NK cells with anti-NKG2D mAb in the presence or absence of IL-27. Presence of IL-27 significantly augmented the expression of *Nrf2* (Fig. 7D). Collectively, these findings provide a novel insight into the stimulatory functions of IL-27 on NK cells in the trachea and alveolar space of the lungs during influenza infections. Role of these changes should be further explored and could partially be responsible for the reduction in the production of IFN-γ in NK cells from *Ebi3*^−/−^ mice.

**Figure 8:**
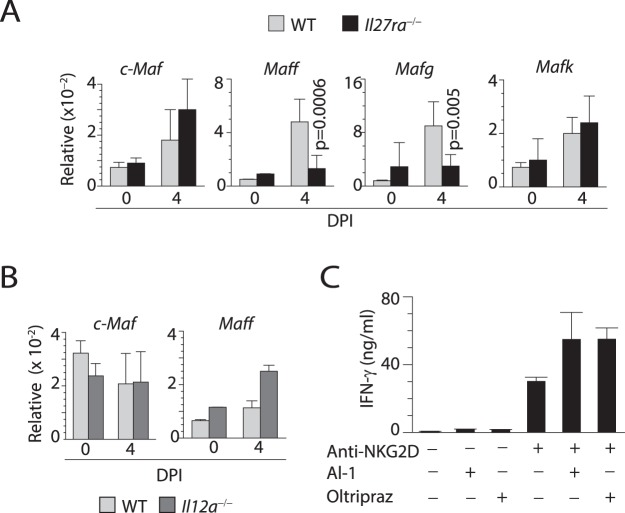
Lack of IL-27Ra but not IL-12Ra results in reduced MAF activation during influenza infection. (**A**) RT-qPCR data shows expression of *cMaf*, *Maff*, *Mafg*, and *Mafk* in sorted NK cells from WT and *Il27ra*^−/−^ (IL-27Rα^−/−^) mice on DPI 4. (**B**) RT-qPCR data shows expression of neither *cMaf* nor *Maff* are altered in sorted NK cells from WT and *Il12ra*^−/−^ (IL-12Rα^−/−^) mice on day four post influenza infection. (**C**) ELISA data showing the production of IFN-γ from NK cells stimulated with anti-NKG2D mAb in the presence or absence of Oltipraz Data in B and C generated from 4 mice per genotype (except mock-infected mice).

Consistent with *Ebi3*^−/−^ mice data, NK cells from *Il27ra*^−/−^ mice (Fig. 8A) but not *Il12a*^−/−^ mice (Fig. 8B) also have reduced expression of MafF. Nrf2 can form a heterodimer with small Mafs, specifically MafF and MafG^54^. To further explore the relevance of reductions in Nrf2 and MafF, we stimulated NK cells using plate-bound anti-NKG2D mAb along with small molecule compounds AI-1 (activates Nrf2 by covalently modifying Keap1, a negative regulator of Nrf2 and Oltipraz that activate anti-oxidant response element, Nrf2^55^. Presence of either AI-1 or Oltipraz enhanced the IFN-γ generation following NKG2D stimulation (Fig. 8C) validating the ability of IL-27 activating a proinflammatory cascade via MafF/MafG-Nrf2 pathways.

## Discussion

NK cells provide the early line of defense against viral infections^56–58^. NK cells execute effector functions through their non-clonotypic activating receptors such as NKG2D, NCR1, and Ly49D. Cytokines are required to regulate the initiation, amplification, and the maintenance of transcriptional memory of an immune response^59^. Cytokines also play a crucial role in establishing ‘immunological priming’. This phenomenon has been well-documented for over two decades for B, and T cells that form the basis for the adaptive immunity^60,61^. However, the occurrence of similar functional adaptations for NK cells is not yet fully understood. Myeloid cell-derived cytokines such as IL-12, IL-15, IL-18, and IL-27 coordinate the receptor-mediated activation of NK cells^62–64^. While the intricate temporal relationship between these cytokines and activation receptor-mediated stimulation of NK cells, *in vivo*, remains a paradox.

Whether and how the IL-12 family of cytokines plays an essential role in transcriptionally-priming NK cells are of active investigations^39^. IL-12 transcriptionally-prime both T and NK cells^65,66^. In T cells, cytokines function as a ‘*third signal’* along with the activation receptor (*primary signal*) and co-stimulation (*second signal*)^67–69^. Although IL-2, IL-15, IL-12, and IL-18 are being widely used to expand and activate NK cells, their *in vivo* role in coordinating the activation via receptors such as NKG2D or NCR1 is not clear. In this study, we utilized an influenza infection model and examined the role of IL-12 cytokine family in coordinating the activation of NK cells, which trafficked into the alveolar space (BAL) and lung tissue within 2–4 days post-infection. Entry of NK cells into the lumen-side of the trachea coincided with the destruction of epithelial cell layer^40,45^. Importantly, a significant percentage of NK cells in the lung tissue produced IFN-γ compared to that of spleens between DPIs 2–4, within the same mice. However, expression of CD107a (Lamp1) a surrogate marker for the release of the enzyme, granzyme B, from the cytotoxic vesicles peaked between DPIs 4–7. Through our earlier work, we showed that this increase in cytotoxic granules coincided with significant damage to the epithelial cell layer between DPIs 4–7^40^. Damage to the ciliary structure of the epithelial cells and an injury to the epithelial layer can make patients susceptible to secondary bacterial infections leading to developing pneumonia and death. Therefore, an efficient immune response to clear the pathogens and rapid regeneration of the epithelial layer is essential to re-establish this protective barrier.

Analyses of transcripts encoding *Il12p35*, *Il12p40*, *Il23p19*, and *Il27p28* indicated that IL-27 was produced during the early phase of influenza infection between DPIs 4–7 both in the alveolar space and lung tissues. IL-27 is one of the IL-12 family members and a proinflammatory cytokine^70–72^. Further analyses demonstrated that a Ly6G^+^ myeloid population was the predominant cell type that produced IL-27 as described earlier^43^. We predict that these Ly6G^+^ myeloid cells are neutrophils and the support for this notion is coming from the findings that human neutrophils along with monocytes/macrophages in patients with melioidosis, a severe form of septicemia caused by the gram-negative bacterium, *Burkholderia pseudomallei*^73^. Production of IL-27 concurred with the expression of IL-27Rα (Wsx1) in NK cells on DPI 4 both in the alveolar space (BAL) and within the lung tissues. Earlier studies have demonstrated that Il-27 reduced lung inflammation by dampening neutrophil recruitment and T_H_1 and T_H_17 functions during influenza infection^43^. However, compared our study that focuses on the early phase of infection, this study explored the role of IL-27-induced IL-10 on T cells at DPI 8^43^.

Myeloid populations including dendritic cells produce IL-12, IL-18, and IL-27 that can function as the ‘third’ signal to prime effector lymphocytes^74,75^. In CD8^+^ T cells, IL-12 can open *Ifng* gene open along with select few hundred genes by chromatin remodeling, relieving gene repression, and allowing continued transcription by promoting augmented histone acetylation^74^. IL-12 along with IL-18 can promote the production of IFN-γ in T and NK cells^76–78^. However, the role of these cytokines in transcriptionally-priming NK cells and to augment NKG2D-dependent effector functions has not been investigated. Our data strongly suggest the presence of IL-27 during the activation of NK cells via NKG2D or Ly49D significantly augmented the production of IFN-γ, GM-CSF, RANTES, and MIP-1α. However, a similar additive effect was not observed when NK cells were stimulated with IL-27 along with IL-12 or IL-18, demonstrating a unique transcriptional and functional role of IL-27. Similarly, IL-6, another proinflammatory cytokine that shares its receptor subunit with the IL-27 receptor, did not augment IFN-γ production either alone or along with anti-NKG2D mAb-mediated activation of NK cells. Thus, it is possible that during an acute viral infection, neutrophils and monocytes/macrophages may provide the ‘third’ signal to NK cells at the site of infection through the production of IL-27. This function may be distinct from the role of dendritic cells that produce both IL-12 and IL-18 within the secondary lymphoid organs (draining lymph nodes) that is to provide a full-fledged activation of NK cells to mediate effector functions. Detailed transcriptomic and genomic analyses are required to further define the temporal and independent roles of these cytokines and their producers.

Earlier reports have suggested that IL-27 can selectively regulate NK cell subsets in human^33^. Through this study, we have found that IL-27 regulates the development of CD27^+^CD11b^+^ NK cell subset. Based on CD27 and CD11b expression, different organs of the body has unique NK cells subset repertoire^49^. Lung NK cells are largely CD27^−^CD11b^+^, whereas BM NK cells are mainly CD27^+^CD11b^−^. These subsets define maturation stages of NK cells^49,79,80^. CD27^high^CD11b^low^ define the earliest stage of NK cells maturation. Our data show that after influenza infection CD27^+^CD11b^+^ effector NK cells appear in the alveolar space and lung tissue of WT mice but not in *Il27ra*^−/−^ mice, suggesting a critical role for IL-27 in regulating this NK subset. We found a defect in CD27^+^CD11b^+^ population; however, no changes were seen in the expression of NK cells activating or inhibitory receptor in the BM of naïve *Il27ra*^−/−^ mice. It is possible that myeloid cells in the BM constitutively produce a low level of IL-27 to regulate NK cells maturation, thereby modulates NK effector function. It could also be possible that CD27^+^CD11b^+^ subset may be recruited to lung tissue from BM after influenza infection. It has also been shown that CD27 expression on NK cells determines migratory capacity and thus IL-27 may play a selective role in promoting the trafficking of a subset of NK cells to the site of infection^49,81^.

Lack of IL-27Rα (Wsx1) or IL-27 subunit EBI3 resulted in impaired NK cell-mediated effector functions and ineffective clearance of influenza infection. In line with earlier work using influenza infection on *Il27ra*^−/−^ mice^43^, we found similar weight loss and mortality trend in *Ebi3*^−/−^ mice. Mixed chimera experiments using splenocytes from WT (CD45.1) and *Il27ra*^−/−^ (CD45.2) further provide strong support that the reduced production of IFN-γ in NK cells from *Il27ra*^−/−^ mice are cell-intrinsic. Our study strongly suggests that IL-27 plays a crucial role during the early phase of influenza infection (DPI 1–4). After DPI 5, the requirement for IL-27 decreased. On DPI 7, the absolute numbers of NK cells and their effector responses were comparable in WT and *Ebi3*^−/−^ or *Il27ra*^−/−^ mice, suggesting a possible role of other cytokines/factors in regulating NK cells functions. It is also important to note, the adaptive immune response by T cells against infected epithelial cells and the B cells producing neutralizing antibody start to occur after DAP 7. Indeed, IL-27 is known to regulate cytoprotective IL-10 generation from T cells^43,82^. NK cells from CD27^−/−^ mice develop phenotypically normal with a similar or enhanced expression of activating or inhibitory receptor^81^. This observation is in line with our *Il27ra*^−/−^ mice data, where there is no defect in NK cells activating or inhibitory receptor expression. Thus, IL-27 plays an essential role in the early NK cell-mediated functions as well as regulates adaptive immune response determining the pathophysiological outcome of influenza infection.

Transcriptional priming of effector lymphocytes by cytokines lead to genomic imprinting, resulting in unique cell fate decisions, and distinct functional outcomes. IL-12 mediates such prototypical chromatin modifications on signature cytokine genes such as *Ifng* and *Il4* in T cells primarily via signal transducer and activator of transcription 4 (STAT4)-dependent chromatin remodeling^83,84^. Earlier studies have shown that the interaction of IL-27 with IL-27Rα/gp130 heterodimer leads to the recruitment of Jak1, Jak2, Tyk2 triggering the phosphorylation of STAT1, STAT2, STAT3, and STAT5 in CD4^+^ T cells^85,86^. STAT1/STAT3 heterodimers translocate into the nucleus, binds to *Tbx21* (T-bet) promoter, increases T-bet levels, which in turn initiates the transcription of *Ifng* gene. *Ifng* gene promoter is known to contain at least three T-bet, two NF-AT, two NF-κB, one AP-1, and one CREB/ATF-2 binding sites. Thus, a substantial reduction in NF-AT, NF-κB, and T-bet can account for the significant reduction in IFN-γ production in NK cells from *Ebi3*^−/−^ or *Il27ra*^−/−^ mice.

Given these premier roles of STAT proteins in the transcription of signature cytokines are well-established, we next focused on the role of IL-27 on Maf proteins. We found that NK cells from *Ebi3*^−/−^ as well as *Il27ra*^−/−^ mice have reduced MafF expression with the highest fold change during influenza infection. Maf-F belongs to small Maf family which are basic-region and basic-leucine zipper (bZIP)-type transcription factors. Nuclear factor E2–related factor 2 (Nrf2) heterodimerizes with small bZIP protein, Maf-F, and activates specific gene transcriptions. This notion is confirmed by our findings that the expression Nrf2 is also reduced in *Ebi3*^−/−^ mice, suggesting IL-27 is also functioning through these transcription factors. These findings are further validated by the earlier observation that Nrf2 indeed interacts with Maf-F and Maf-G^54^. In summary, our findings demonstrate the crucial role played by IL-27 during the early phase of influenza infection. IL-27 plays a central role in augmenting the stimulation via NKG2D and NCR1. Future studies are warranted to define the temporal and functional relationship of IL-27 with IL-12, IL-18, and IL-15.

## Methods

### Mice and stable cell lines

C57BL/6, *B6*.*SJL* (*H-2*^*b*^ *CD45*.*1*^+^), *Il12a*^−/−^ mice and *Ebi3*^tm1Rsb^ (*Ebi3*^−/−^) mice were purchased from Jackson Laboratory (Bar Harbor, ME) and maintained in pathogen-free conditions at the Biological Resource Center at the Medical College of Wisconsin. *Rag2*^−/−^γ*c*^−/−^ mice were purchased from Jackson Laboratory (Bar Harbor, ME). Generation of *Il27ra*^−/−^ gene knockout mice has been described earlier^87^. Female and male mice between the ages of 6 and 12 weeks were used. EL4 (ATCC, Manassas, VA), EL4^H60^ (a derivative of EL4 and was generated by our laboratory), RMA/S (a kind gift from Dr Nilabh Shastri, UC Berkeley), and YAC1 (ATCC, Manassas, VA) cells were maintained in RPMI-1640 medium containing 10% heat-inactivated FBS (Life Technologies, Carlsbad, CA). Those cell lines were periodically tested to exclude the possibility of mycoplasma contamination. The generation of stable H60-expressing EL4 cell lines has been described^88^.

### Ethics Statement

All animal experiments in this study were conducted in accordance with the guidelines of the US Government Animal Welfare Act (AWA) 7 U.S.C. § 2131. Institutional Animal Care approved all animal protocols and Use Committees of the IACUC at the Medical College of Wisconsin, Milwaukee, WI. Medical College of Wisconsin is formally accredited by AAALAC and all the animal care and use-protocols used in this study fully adhere to the specified guidelines of AAALAC. The unique animal protocols that are approved by the IACUC and used in this study are AUA1500 and AUA1512.

### Reagents

Recombinant IL-27p28 was purchased from Shenandoah Biotechnology Inc (Warwick, PA). Al-1 and Oltipraz were purchased from Sigma-Aldrich (St. Louis, MO).

### *In vivo* infection

WT mice 6–8 weeks of age were deeply anesthetized and were intranasally challenged with 500 plaque-forming unit of PR8 virus in sterile phosphate-buffered saline in a total volume of 30 μl through one nostril as described^40^. Mock infections were carried out using only sterile phosphate-buffered saline without the virus. After infections, mice were observed for weight loss and mortality for two weeks.

### BAL fluid collection

Mice were euthanized, and thoracic cavity was cut open, and a 1 cm incision was made parallel to the trachea through the fur of the mouse to expose it. A midline incision was made on the ventral aspect of the trachea slightly above the thoracic inlet 0.3 ml of phosphate-buffered saline-1% bovine serum albumin was infused into the lung through the thoracic inlet using a sterile 1 ml syringe. Lavage fluid was aspirated, aliquoted, and frozen until use.

### Antibodies

Anti-NK1.1 (PK136), anti-NKG2D (A10), CD27 (LG.7F9), CD11b (M1/70), Ly6G (RB6-8C5) LAMP1 (4D1B), IFNγnti-NK1.1 (PK136), anti-NKG2D (A10), CD27 (LG.7F9), CD11b (M1/70), Ly6G (RAnti-Ly49A (A1), Anti-Ly49D (4E5), anti-Ly49C/I (SW5E6), and anti-Ly49G (4D11) were obtained from BD PharMingen, (San Diego, CA). Anti-NCR1 (MAB2225) were from R&D Systems (Minneapolis, MN).

### NK cell preparation

Single-cell suspensions from spleen were passed through nylon wool columns for depletion of adherent populations consisting of B cells and macrophages. Cells that did not adhere to nylon wool were cultured with 1000 U/ml of IL-2 (NCI-BRB-Preclinical Repository, Bethesda, DC). The purity of the NK cultures was checked, and preparations with more than 95% of NK1.1^+^ cells were used on day 7 for *in vitro* experiments.

### Lymphocyte preparations

Lungs were minced into pieces and digested with 500 μl of 10 mg ml^−1^ collagenase (C5138, 100 mg, Sigma-Aldrich, St Louis, MO) and 50 μl of 200 IU of DNase I from bovine pancreas (D4527-20KU, Sigma-Aldrich, St Louis, MO) in total volume of 5 ml of RPMI1640 complete medium for 1 h at 37 °C. Lymphocytes from the lung were isolated by Ficoll–Hypaque density gradient (Sigma-Aldrich, St Louis, MO).

### Flow cytometry

Single-cell preparations from spleen, lung, and BAL lymphocyte were surface stained with CD3ε (145-2C11), NK1.1 (PK136) and NCR1 (29A1.4) (eBioscience, San Diego, CA). For intracellular staining, single-cell suspensions from lung or BAL or spleen or trachea were the first stain for the surface receptor for 30 min followed by fixation (Fixation buffer, Bio Legend, San Diego, CA), and permeabilization (permeabilization wash buffer, Bio Legend) steps for additional 20 min. Cells were stained intracellularly with phycoerythrin-indo tricarbocyanine (PE-TR)–conjugated mAb to IFN-γ (XMG1.2; eBioscience, San Diego, CA) in permeabilization wash buffer and analyzed in LSR-II (BD Biosciences, San Jose, CA).

### Adoptive transfer

Single-cell suspensions from the splenocytes of B6.SJL (H-2^b^ CD45.1^+^) and *Il27ra*^−/−^ (H-2^b^, CD45.2^+^) mice were mixed 1:1 and adoptively transferred into *Rag2*^−/−^γ*c*^−/−^ (CD45.2^+^) intravenously as described earlier^6^. Donor CD45.1^+^ and CD45.2^+^ NK cells were detected, and the generation of IFN-γ was analyzed by flow cytometry following day two post adoptive transfer.

### Cytotoxicity assays

WT, *Ebi3*^−/−^ and *Il27ra*^−/−^ derived NK cell-mediated cytotoxicity against EL4 cells, EL4^H60^, RMA-S and YAC1 cells was quantified by ^51^Cr-release assays at a varied ratio of effector to target cells^89^. Cytotoxicity was also performed in the presence or absence of recombinant IL-27p28 (10 ng/ml, Shenandoah Biotechnology, Warwick, PA). Specific lysis was calculated by the amount of absolute, spontaneous and experimental release of ^51^Cr from target cells.

### Quantification of cytokines and chemokines

NK cells from WT, *Ebi3*^−/−^, and *Il27ra*^−/−^ were cultured in IL-2, Fc receptors were blocked with mAb to CD16-CD32 (2.4G2; BD Pharmingen) and cells were activated for 18 h with plate-bound anti-NKG2D (A10; eBioscience) or Ly49D (4E5; BD Pharmingen, San Jose CA) mAbs in presence or absence of recombinant IL-27 (10 ng/ml). Culture supernatants were analyzed by Bioplex assay (Bio-Rad, Hercules CA) as described^6^. NK cells that had been cultured in IL-2 were also treated with IL-12 (1 ng/ml; R&D Systems, Minneapolis, MN) or IL-18 (10 ng/ml; R&D Systems, Minneapolis, MN) in the presence or absence of recombinant IL-27 (10 ng/ml) for 18 h, and the supernatants were analyzed similarly.

RT-qPCR. For quantification of *Ifng* mRNA, sorted NK cells from influenza-infected mice lysed, and total RNA was purified with an RNeasy Mini Kit (Qiagen). Real-time PCR was done with SYBR green protocol and an ABI7900 HT thermal cycler. Transcripts in each sample were assayed in triplicate, and the mean cycling threshold was used for calculation of the change in expression. The control (housekeeping) gene *Gapdh* (5′ CCT GCA CCA CCA ACT GCT TAG 3′, sense and 5′ GTG GAT GCA GGG ATG ATG TTC 3′, anti-sense) was used for global normalization in each experiment. Primer sequences for *Il12p35:* 5′ CCG GTC CAG CAT GTG TCA A 3′ (sense) and 5′ CAG GTT TCG GGA CTG GCT AAG A 3′ (anti-sense); *Il12p40:* 5′ ACT CAC ATC TGC TGC TCC ACA AG 3′ (sense) and 5′ CAC GTG AAC CGT CCG GAG TA 3′ (anti-sense); *Il23p19:* 5′ TGT GCC CCG TAT CCA GTG T 3′ (sense) and 5′ CGG ATC CTT TGC AAG CAG AA 3′ (anti-sense); *Il27p28:* 5′ ATG TCC ACA GCT TTG CTG AAT CT 3′ (sense) and 5′ CTG CAG CCA GCA CCT GAA AG 3′ (anti-sense); *Ifng:* 5′ GAC TGT GAT TGC GGG GTT GT 3′ (sense) and 5′ GGC CCG GAG TGT AGA CAT CT 3′ (anti-sense); *Nfe2*: 5′ CCT GCT GTG ACT CCA CCA CA 3′ (sense) and 5′ GCC AGA GTC TGG TCC AGG TTC 3′ (anti-sense); *Nrf1*: 5′ TTG GAA CAG CAG TGG CAA GA 3′ (sense) and 5′ CTC ACT TGC TGA TGT ATT TAC TTC CAT 3′ (anti-sense); *Nrf2*: 5′ CTC GCT GGA AAA AGA AGT G 3′ (sense) and 5′ CCG TCC AGG AGT TCA GAG G 3′ (anti-sense); *Nrf3*: 5′ GCA GGA GGA AAA CGA GGA A 3′ (sense) and 5′ GAC CAA TGT AGA TGG CTC TCG 3′ (anti-sense); *c-Maf*: 5′ ACT GAA CCG CAG CTG CGC GGG GTC AG 3′ (sense) and 5′ CTT CTC GTA TTT CTC CTT GTA GGC GTC C 3′ (anti-sense); *Maff*: 5′ AGC GTC ATC ACC ATC GTC AA 3′ (sense) and 5′ GTC ACT ACA ACG AGT GGC AGA 3′ (anti-sense); *Mafg*: 5′ CGA GAG TTG AAC CAG CAC CT 3′ (sense) and 5′ GCA ACT GCA CGT GTC CCT AT 3′ (anti-sense); *Mafk*: 5′ TAG CCG CGT AGC CTC TGT T 3′ (sense) and 5′ CCC CTG TTC TTA GCG ATG ATG 3′ (anti-sense); *Nqo1*: 5′ AGC CAA TCA GCG TTC GGT A 3′ (sense) and 5′ GAA TGG GCC AGT ACA ATC AGG 3′ (anti-sense); *Gclc*: 5′ CTA CCA CGC AGT CAA GGA CC 3′ (sense) and 5′ CCT CCA TTC AGT AAC TGG AC 3′ (anti-sense); *Tfam*: 5′ GTC GCA TCC CCT CGT CTA TC 3′ (sense) and 5′ GCT GGA AAA ACA CTT CGG AAT AC 3′ (anti-sense); *Cpt1a*: 5′ TGG CAT CAT CAC TGG TGT GTT 3′ (sense) and 5′ GTC TAG GGT CCG ATT GAT CTT TG 3′ (anti-sense).

### Experimental data and statistical analysis

A paired, two-sample Student’s *t*-test was used for statistical analysis, with equal or unequal variance, depending on the type of data. *P* values of 0.05 or less were considered significant. Normal distribution of sample variance was assumed by earlier studies from other laboratories with data sets similar to ours.

## Supplementary information

Supplementary Figure 5
