# Supplementary material for: IL-27 promotes NK cell effector functions via Maf-Nrf2 pathway during influenza infection
Source: Sci Rep. 2019 Mar 21;9:4984. doi: 10.1038/s41598-019-41478-6 (PMC6428861; doi:10.1038/s41598-019-41478-6)
Supplement: Supplementary file 1 — Supplementary Figure 5 [file 41598_2019_41478_MOESM1_ESM.pdf]

Supplemental Information

IL-27 promotes NK cell effector functions via Maf-Nrf2 pathway during influenza infection

Pawan Kumar[1], Kamalakannan Rajasekaran[1], Arash Nanbakhsh[1], Jack Gorski[2], Monica S Thakar[1,3] and Subramaniam Malarkannan[1,3-5,¥]

[1]Laboratory of Molecular Immunology and Immunotherapy, [2]Laboratory of Molecular Genetics, Blood Research Institute, 8727 Watertown Plank Road, Milwaukee, WI 53226.

Departments of [3]Pediatrics, [4]Microbiology & Immunology, [5]Medicine, Medical College of Wisconsin, Milwaukee, WI 53226,

[¥] To whom correspondence should be addressed (Subra.malar@bcw.edu).

Supplemental Figures

Supplemental Figure 1. Production of IL-27p28 during influenza infection. A) IL-27p28 is produced by cells in the BAL during influenza infection that peaks on DPI 4 (top panels). IL-27p28 is predominantly produced by Ly6G-positive cells in the BAL (bottom panels). **B**) IL-27p28 is produced by cells within the lung tissue during influenza infection and appears to be distinct from that of BAL-derived cells. Data shown in A and B are FACS plots of cells from BAL or lung tissues from influenza virus-infected mice intracellularly stained with anti-IL-27p28 antibody. Data presented are a representative of four different mice for each DPI and from two independent experiments.

Supplemental Figure 2. Absolute number of NK cells responding to influenza infection is significantly reduced in *Il27ra*$^{-/-}$ mice. A) CD3ε^{-}NK1.1^{+} NK cells were gated and analyzed for intracellular IFN-γ or **B**) the surface expression of CD107a (LAMP1) from BAL or lung tissues of infected mice. Data in A and B are the average and standard deviation of three mice each from two independent experiments.

Supplemental Figure 3. Lack of IL-12α does not affect the effector functions of NK cells during influenza infection. A) Intracellular IFN-γ or surface LAMP expression in WT and *Il12a*$^{-/-}$ CD3ε^{-}NK1.1^{+} NK cells isolated from different days post infection. **B**) Data shown are the average percentages of IFN-γ-positive CD3ε^{-}NK1.1^{+} NK cells from BAL and lung tissues of infected WT or *Il12a*$^{-/-}$ mice. **C**) Expression of *Ifng* mRNA in sorted CD3ε^{-}NK1.1^{+} NK cells from WT and *Il12a*$^{-/-}$ NK cells on day 0 and day four post influenza infection presented relative to that of the control gene *Gapdh*.

Supplemental Figure 4. NK cell development is largely unaltered in *Il27ra*$^{-/-}$ mice. A) CD27 and CD11b subset specification among CD3ε^{-}NK1.1^{+} NK cells in the bone marrow of WT and *Il27ra*$^{-/-}$ mice. **B**) Quantification of NK subsets based on CD27 and CD11b expression in the bone marrow of WT and *Il27ra*$^{-/-}$ mice. **C**) Percentages of inhibitory Ly49A, Ly49C/I, NKG2A, and Ly49G2 expressing NK cells are comparable between the bone marrows of WT and *Il27ra*$^{-/-}$ mice. **D**) Percentages of activating Ly49D and Ly49H expressing NK cells are comparable between the bone marrows of WT and *Il27ra*$^{-/-}$ mice. **E**) Average percentages of different NK cell subsets in the bone marrow of WT and *Il27ra*$^{-/-}$ mice. Data in A-E were generated from three mice per genotype.

Supplementary Figure 5. IL-27 regulates NKG2D-mediated cytotoxicity. **A**) Cytotoxicity of WT, *Ebi3*$^{-/-}$, and *Il27ra*$^{-/-}$ NK cells towards indicated target cells. **B**) Cytotoxicity of NK cells from WT,

Ebi3$^{-/-}$, and *Il27ra*$^{-/-}$ mice that are cultured in IL-2 and tested against indicated target cells in the presence or absence of recombinant IL-27. Data shown in A are a representative graph of 2-3 independent experiments with n=2-3 mice in each group performed in triplicates. Data shown in B are generated with 3-6 WT, 3-5 *Ebi3*$^{-/-}$, or 3 *Il27ra*$^{-/-}$ mice with and without rIL-27 stimulation (10 ng/ml).

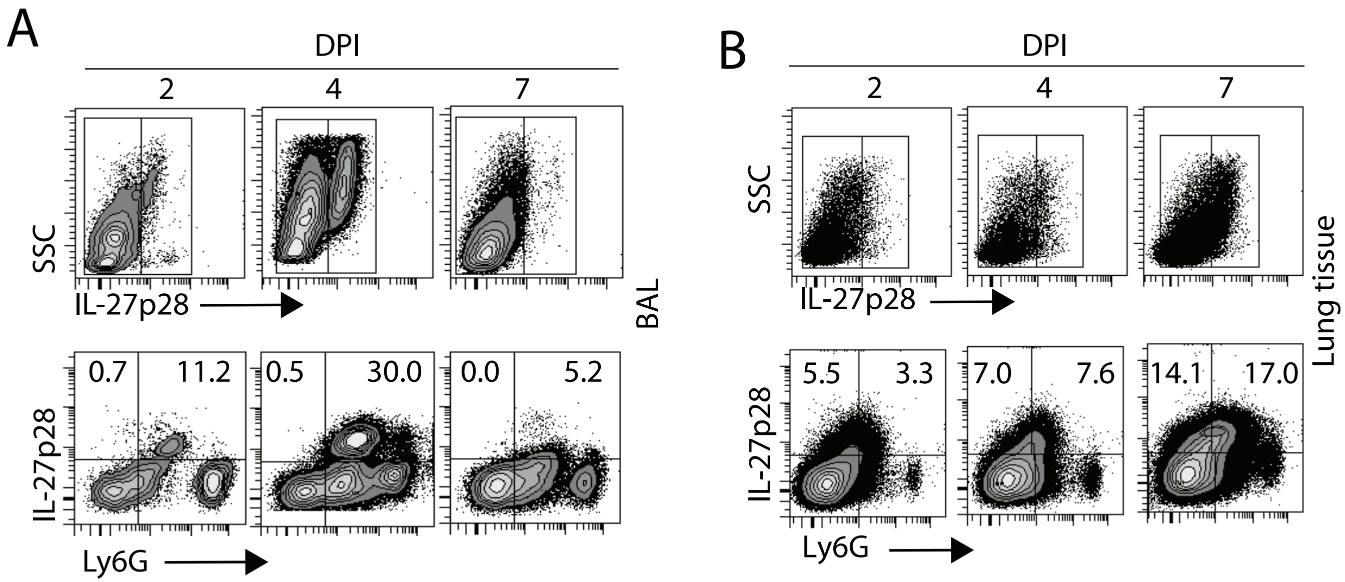
A
DPI
2
4
7
SSC
IL-27p28
BAL
IL-27p28
0.7
11.2
0.5
30.0
0.0
5.2
Ly6G
B
DPI
2
4
7
SSC
IL-27p28
Lung tissue
IL-27p28
5.5
3.3
7.0
7.6
14.1
17.0
Ly6G

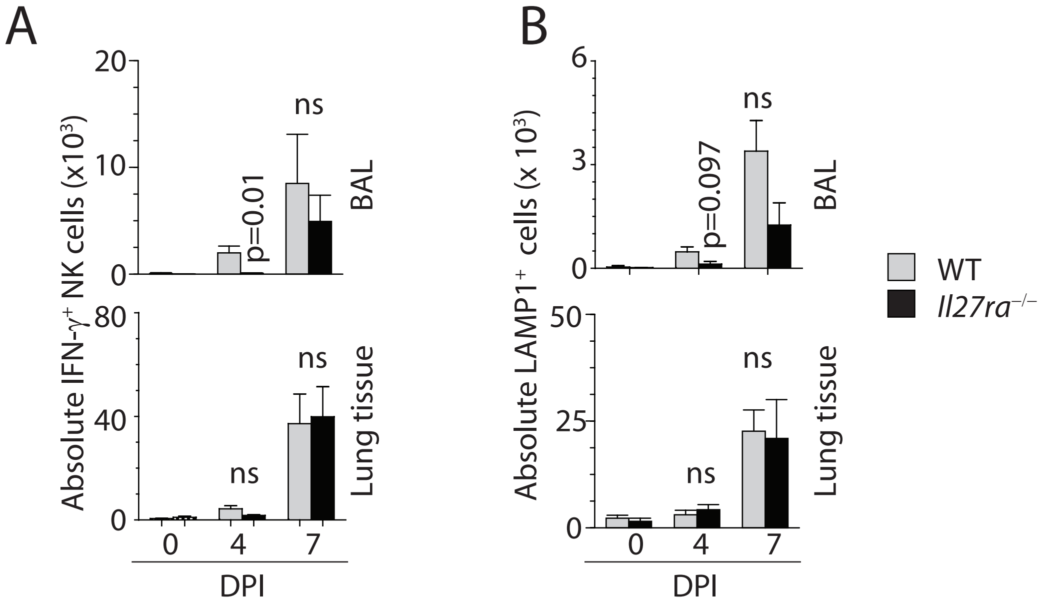
A
Absolute IFN-γ+ NK cells (x10³)
20
10
0
80
40
0
p=0.01
ns
ns
ns
BAL
Lung tissue
0
4
7
DPI
B
Absolute LAMP1+ cells (x 10³)
6
3
0
50
25
0
p=0.097
ns
ns
ns
BAL
Lung tissue
0
4
7
DPI
WT
Il27ra−/−

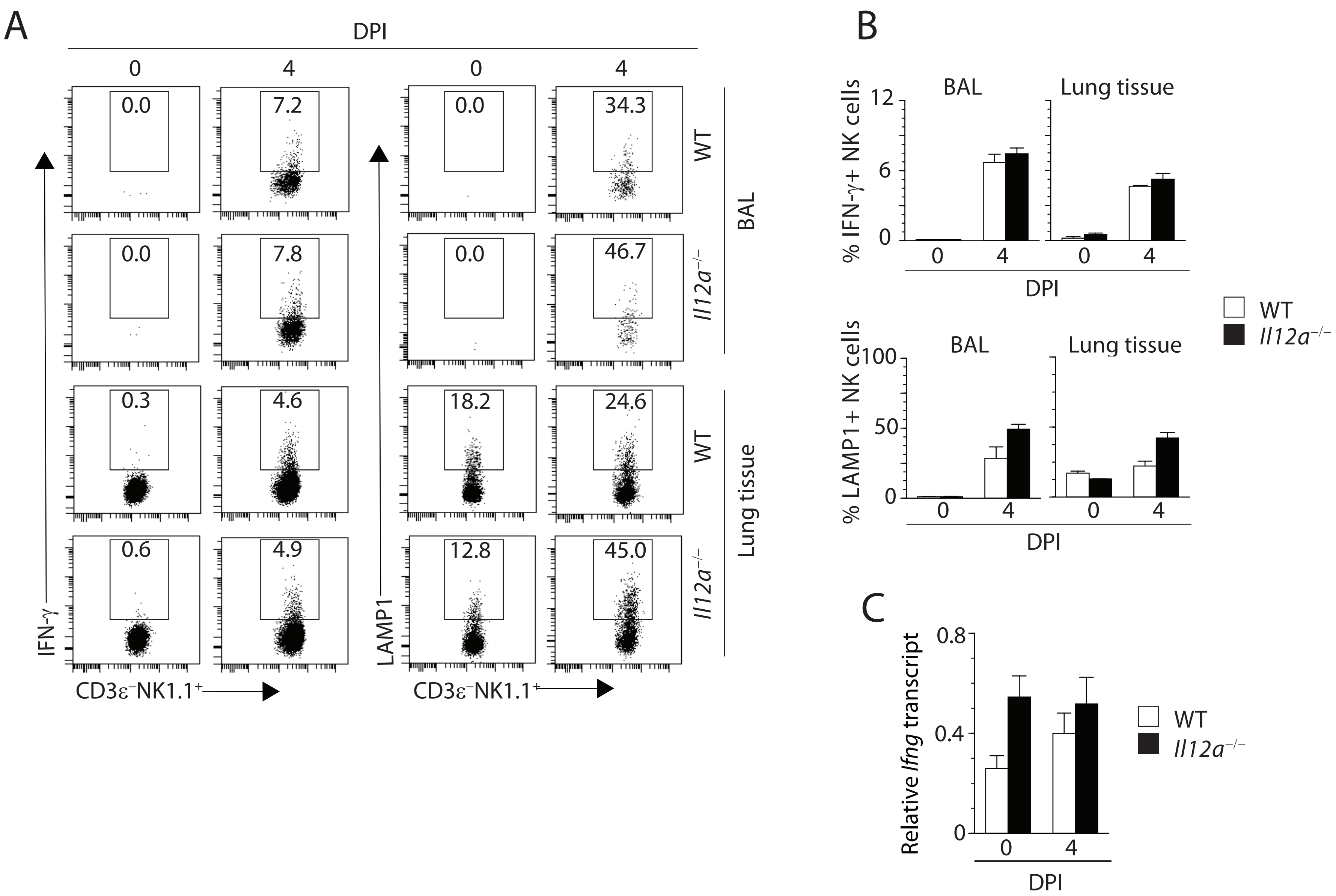

A
DPI
0
4
0
4
0.0
7.2
0.0
34.3
0.0
7.8
0.0
46.7
0.3
4.6
18.2
24.6
0.6
4.9
12.8
45.0
WT
Il12a−/−
BAL
WT
Il12a−/−
Lung tissue
IFN-γ
LAMP1
CD3ε−NK1.1+
CD3ε−NK1.1+
B
BAL
Lung tissue
% IFN-γ+ NK cells
12
6
0
0
4
0
4
DPI
WT
Il12a−/−
BAL
Lung tissue
% LAMP1+ NK cells
100
50
0
0
4
0
4
DPI
C
Relative Ifng transcript
0.8
0.4
0
0
4
DPI
WT
Il12a−/−

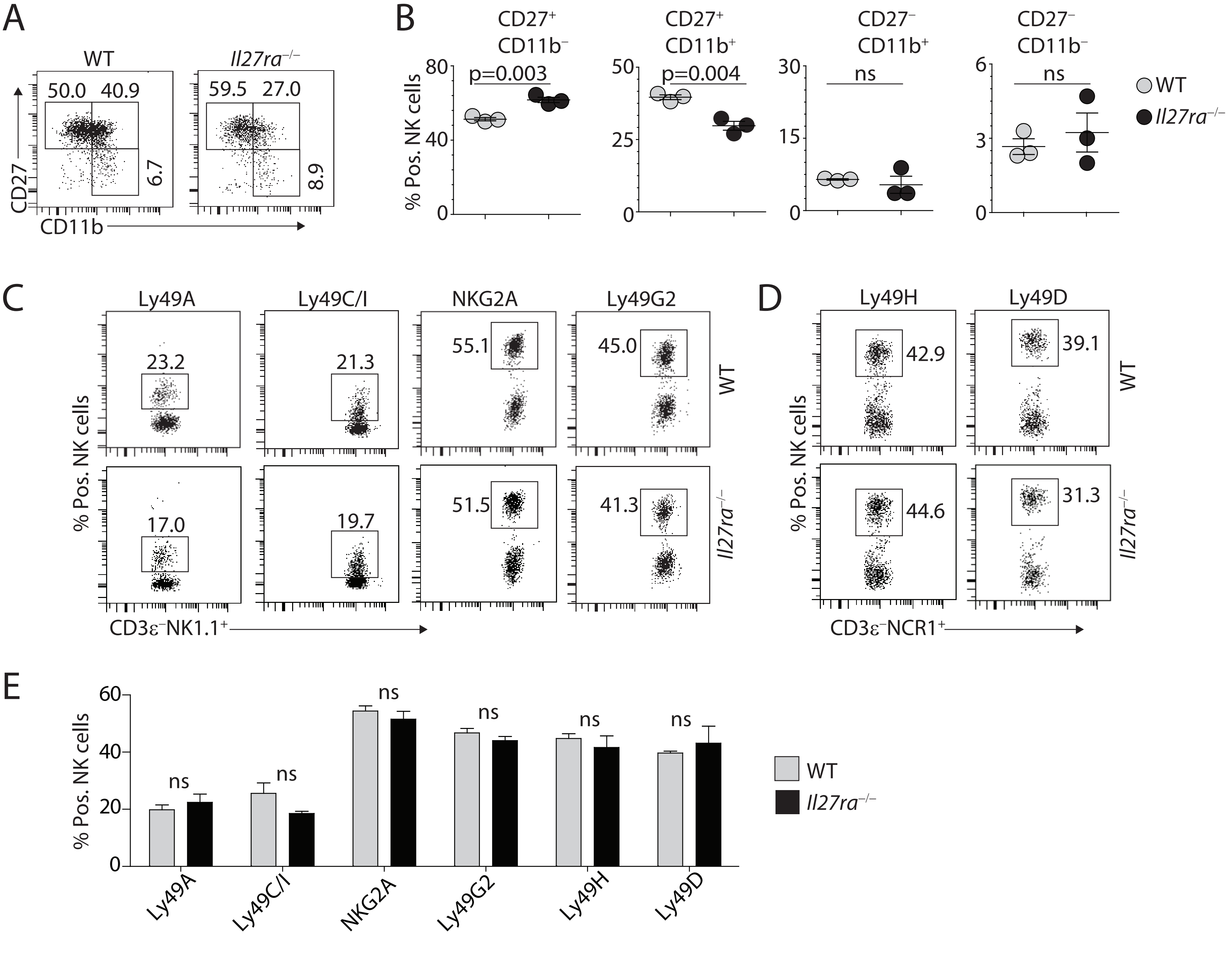
A
WT
Il27ra−/−
50.0 40.9
6.7
59.5 27.0
8.9
CD27
CD11b
B
CD27+ CD11b−
CD27+ CD11b+
CD27− CD11b+
CD27− CD11b−
p=0.003
p=0.004
ns
ns
% Pos. NK cells
WT
Il27ra−/−
C
Ly49A
Ly49C/I
NKG2A
Ly49G2
23.2
21.3
55.1
45.0
17.0
19.7
51.5
41.3
% Pos. NK cells
CD3ε−NK1.1+
D
Ly49H
Ly49D
42.9
39.1
44.6
31.3
% Pos. NK cells
CD3ε−NCR1+
E
% Pos. NK cells
ns
Ly49A
Ly49C/I
NKG2A
Ly49G2
Ly49H
Ly49D
WT
Il27ra−/−

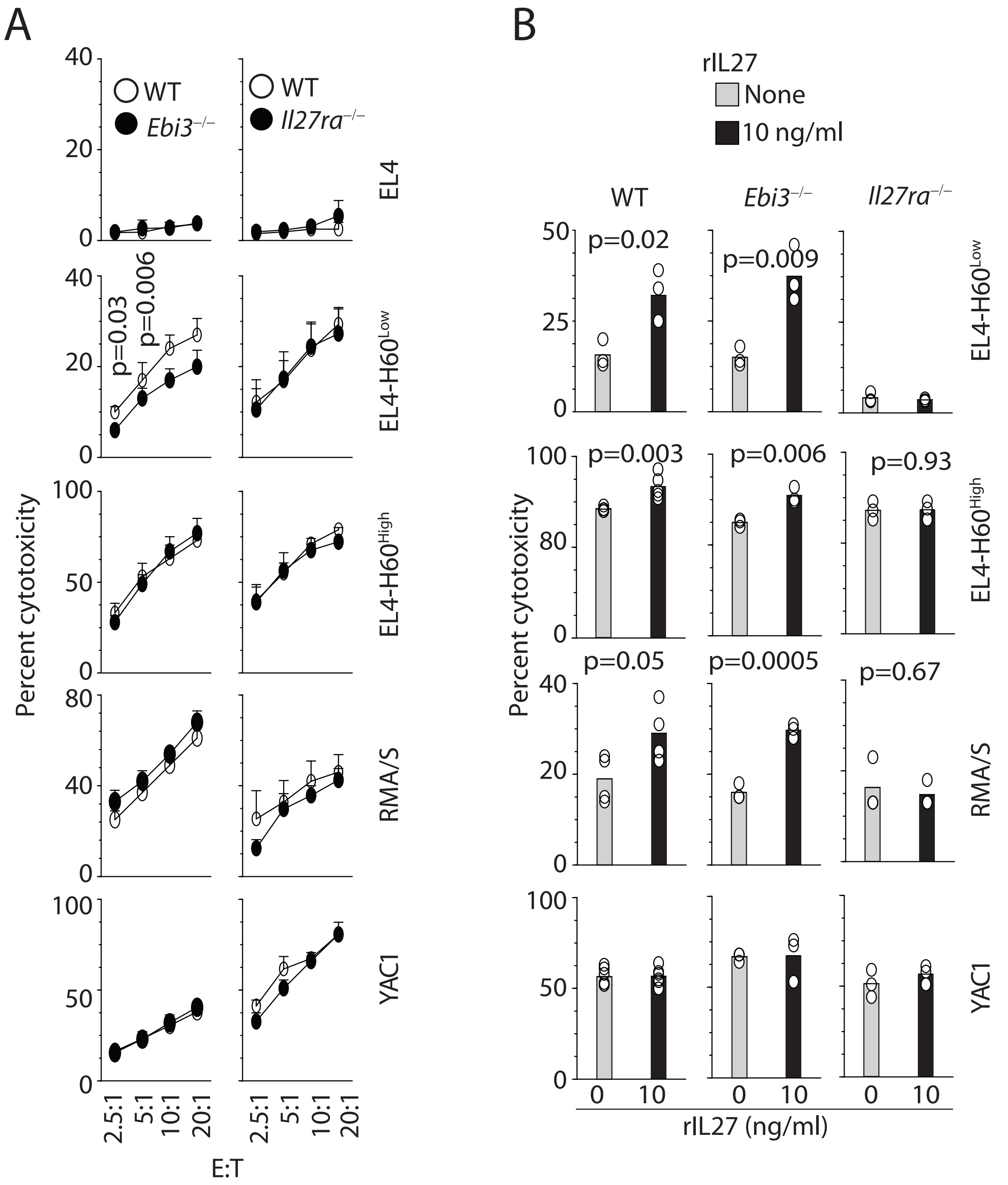
A
WT
Ebi3−/−
WT
Il27ra−/−
EL4
EL4-H60Low
EL4-H60High
RMA/S
YAC1
p=0.03
p=0.006
Percent cytotoxicity
2.5:1
5:1
10:1
20:1
E:T
B
rIL27
None
10 ng/ml
WT
Ebi3−/−
Il27ra−/−
p=0.02
p=0.009
p=0.003
p=0.006
p=0.93
p=0.05
p=0.0005
p=0.67
rIL27 (ng/ml)
